# The meaning of work for teachers in educational institutions in the department of magdalena

**DOI:** 10.1192/j.eurpsy.2024.1714

**Published:** 2024-08-27

**Authors:** K. L. Perez Correa, L. P. Pedraza Àlvarez, J. Viloria Escobar, J. J. Gómez Rangel

**Affiliations:** ^1^Magdalena, Universidad Cooperativa de Colo; ^2^Magdalena, Universidad del Magdalena, Santa Marta; ^3^Magdalena, Instituto Nacional de Formación Técnica Profesional Humberto Velásquez García, Cienaga, Colombia

## Abstract

**Introduction:**

The psychosocial and mental health-oriented variables of people are determinants for their life in society and their roles within organizations, especially educational institutions that are endowed with social complexities.

**Objectives:**

The objective of this research was to understand the meaning of work for teachers in educational institutions in the department in order to recognize elements such as the level of importance that work holds for them and the factors that either promote or hinder that centrality.

**Methods:**

This is a descriptive study with a quantitative methodology, and the sample selection was done for convenience, taking into account ethical aspects such as the handling of confidentiality for both the individuals who participated in this study and the educational institutions involved.

**Results:**

Regarding the meaning attributed to work by teachers, the results indicate that 29.6% of teachers declare themselves neutral when it comes to the statement that “the most important things in people’s lives are related to work”. 26.8% of teachers are neutral regarding the statement that “the primary function of work is to generate income”, and 17.9% somewhat agree. 20.7% disagree to some extent. 15.6% disagree with the statement that “people’s primary goals in life should be oriented toward work”. 25.1% of teachers are neutral, and 17.9% somewhat agree with the statement that “the main function of work is to enable interesting contacts with other people”. Only 11.7% strongly agree with the statement that “work is, in general, one of the most important things in people’s lives.

**Conclusions:**

It is concluded that there is a need to implement strategies that contribute to the strengthening of the teaching profession and contribute to improving educational quality

**Disclosure of Interest:**

None Declared

